# Infantile Convulsions

**Published:** 1889-09

**Authors:** 


INFANTILE CONVULSIONS—A FACT WORTH KNOWING.—Dr. T. J. Heard says: ''In nineteen cases out of twenty, infantile spasms or convulsions may be arrested in one minute by the application of one or two dry cups on the back, from the seventh cervical to the first dorsal vertebra. This will secure a remission, during which emetics, purgatives, or anything else that the indications may require, may be used.

